# Limited Genetic Structure of Gypsy Moth Populations Reflecting a Recent History in Europe

**DOI:** 10.3390/insects9040143

**Published:** 2018-10-18

**Authors:** Nikola Lacković, Milan Pernek, Coralie Bertheau, Damjan Franjević, Christian Stauffer, Dimitrios N. Avtzis

**Affiliations:** 1 Croatian Forest Research Institute, Cvjetno naselje 41, 10450 Jastrebarsko, Croatia; nikolal@sumins.hr (N.L.); milanp@sumins.hr (M.P.); 2 UMR CNRS-UFC 6249 Chrono-Environment, Université de Franche Comte, 25200 Montbéliard, France; coralie.bertheau@gmail.com; 3 Faculty of Science, University of Zagreb, HR-10000 Zagreb, Croatia; damianf@zg.biol.pmf.hr; 4 Department of Forest and Soil Sciences, BOKU, University of Natural Resources and Life Sciences, A-1180 Vienna, Austria; christian.stauffer@boku.ac.at; 5 Forest Research Institute, Hellenic Agricultural Organization Demeter, Vasilika, 57006 Thessaloniki, Greece

**Keywords:** forest pest, population genetics, population outbreaks, range shift, admixture

## Abstract

The gypsy moth, *Lymantria dispar*, a prominent polyphagous species native to Eurasia, causes severe impacts in deciduous forests during irregular periodical outbreaks. This study aimed to describe the genetic structure and diversity among European gypsy moth populations. Analysis of about 500 individuals using a partial region of the mitochondrial COI gene, *L. dispar* was characterized by low genetic diversity, limited population structure, and strong evidence that all extant haplogroups arose via a single Holocene population expansion event. Overall 60 haplotypes connected to a single parsimony network were detected and genetic diversity was highest for the coastal populations Croatia, Italy, and France, while lowest in continental populations. Phylogenetic reconstruction resulted in three groups that were geographically located in Central Europe, Dinaric Alps, and the Balkan Peninsula. In addition to recent events, the genetic structure reflects strong gene flow and the ability of gypsy moth to feed on about 400 deciduous and conifer species. Distinct genetic groups were detected in populations from Georgia. This remote population exhibited haplotypes intermediate to the European *L. dispar dispar*, Asian *L. dispar asiatica*, and *L. dispar japonica* clusters, highlighting this area as a possible hybridization zone of this species for future studies applying genomic approaches.

## 1. Introduction

The gypsy moth, *Lymantria dispar* L. (Lepidoptera, Erebidae), is a polyphagous pest feeding on more than 400 plant species with a preference for species within the genus *Quercus* [1]. *Lymantria dispar* is native to the Palearctic region [1,2] and became invasive in North America after its intentional introduction for hybridization experiments [3]. Currently, *L. dispar* is considered one of the most notorious forest pests worldwide [4], with outbreaks in its native range being common particularly in the oak forests of the Mediterranean region, while in Asia the broader spectrum of potential hosts renders gypsy moth capable of even more massive outbreaks [5]. Nonetheless, the potential of gypsy moth populations to erupt varies greatly [6] as in cases of recent invasion an outbreak can be sustained [7]. For all these reasons, gypsy moth, has been in the spotlight of various investigations ranging from population dynamics [8] and dispersal ecology [9,10] to the distribution of its genetic diversity at a global scale [11,12]. Three subspecies are recognized throughout the temperate part of the world due to their economic importance; European (*L. dispar dispar*) and two Asian (*L. dispar asiatica* in Russia and Asia and *L. dispar japonica* in Japan), and each of them is described separately based on morphological and behavioral traits [1]. Nonetheless, the status of the two Asian subspecies still remains disputable [13], as recent molecular investigations argue for an even more elaborate pattern among the far east populations [12]. However, female flight capability turned out to be the most important behavioral trait distinguishing Asian subspecies from European [10,14].

First genetic analysis based on DNA sequences resulted in developing an mtDNA marker that verified the close resemblance of North American and European population, that both were distinctly separated from Asian populations [11]. This pattern was confirmed in subsequent studies which showed that North American populations were related to French populations and additionally that Asian population were separated into a western and eastern genetic entity [14], where Japanese populations form a separate cluster differentiated from Western Asian populations [15].

Until recently, studies on European populations of gypsy moth were mostly integrated into broader investigations that aimed at a global overview of this species [14,15]. A study analyzing Croatian populations revealed a genetic split between coastal and continental populations caused by the Dinaric Alps [16]. In this study we sampled 38 locations from sixteen European countries, and additionally two Georgian populations to reveal insight into the phylogeography and demography of this species in Europe.

## 2. Materials and Methods

Samples (larvae) were collected from 36 localities in sixteen European countries and two additional sites from Georgia between 2010 and 2014 (Table 1), with specimens from the same locality/site constituting a geographic population. Larvae were taken from distant trees (not more than one larva per tree) so to avoid any bias induced by the use of mtDNA marker, and after collection they were immediately put into 99% ethanol and stored at −20 °C in laboratory. In total, 497 individuals were analyzed. One pair of abdominal legs was used for genomic DNA extraction from each specimen individually, using SIGMA Aldrich mammalian genomic DNA extraction kit (Sigma, St. Louis, MO, USA) and following manufacturer’s instruction. DNA amplification was performed with primers UEA5 and UEA10 [17] in 20 μL reactions containing 2 mM of MgCl2, 100 mM of dNTPs (Fermentas, Vilnius, Lithuania), 0.5 mM of each primer 1U of Taq (Fermentas, Vilnius Lithuania). Thermocycling consisted of an initial denaturation step at 94 °C (2 min), which was followed by 33 cycles at 94 °C (30 s), 48 °C (60 s), and 68 °C (60 s), and a final extension step at 68 °C (10 min). All reactions were checked for amplification by gel electrophoresis, and confirmed products were purified using peqGOLD Cycle-Pure Kit (PeqLab, Erlangen, Germany) and directly sequenced with UEA10 at Eurofins MWG Operon (Mainz, Germany). Sequences were manually examined using Chromas Lite (Technelysium, Brisbane Australia) and aligned using ClustalW algorithm incorporated in BioEdit [18]. Upon alignment, ends of sequences were trimmed in order to obtain full overlap. Finally, this alignment was used for determination of haplotype sequences, using TCS 1.21 [19]. Singleton sequences were verified by an additional PCR and sequencing. Haplotype sequences were deposited in NCBI with accession numbers: KY628707 to KY628749.

Haplotype diversity, nucleotide diversity, mean number of pairwise differences and allelic richness were calculated for each population using ARLEQUIN [20] and Contrib 1.02 [21]. Parsimony network was reconstructed by cladistic analysis in TCS 1.21 and drawn in CorelDraw X5 (Ottawa, ON, Canada). Ambiguities in the cladogram were resolved by applying topological, geographic, and frequency criteria, where applicable [22], while phylogeographic inferences were assessed with ANeCA v1.2 [23]. Overall population differentiation was assessed by testing if N_ST(obs)_ was greater than N_ST(exp)_ using heuristic permutation test with 1000 replicates in PERMUT 1.0 [24], and subsequently compared with G_ST_ in order to assess the depth of differentiation.

To separate the source of variation in three hierarchical levels (among groups of populations (F_CT_), among populations within groups (F_ST_), and within populations (F_SC_)), objective grouping and Analysis of Molecular Variance were performed using ARLEQUIN [19] based on the host of each population (Table 1). The best-fit nucleotide substitution model of our data was selected among 88 different models (in this case GTR with Gamma distributed heterogeneity of rates) as implemented in jModeltest 2.1.7 [25], and this model was then used in MrBayes 3.2 [26]. Two separate chains were run in the Bayesian analysis, and the initial run lasted 2 × 10^6^ generations, with a sampling frequency of 100 generations in order to define the trees to be discarded in the second run. As a plateau between standard deviations was reached after 5 × 10^5^ generations, 500 trees were discarded from the estimation of the consensus tree. To better understand the assignment of haplotypes in clades and their geographic distribution, we also used sequences deposited in NCBI GenBank from previous investigations of *L. dispar* (HM013724 (Russia, Vostochnyy), HM013736 (Japan), HM013737 (Japan) [15]; KX436205 (Japan, Honshu), KX436223 (Japan, Nagano), KX436230 (Russia, Vladivostok) [27]; KY923059 (Russia, Primorski), KY923063 (Lithuania), KY923064 (Russia, Krasnoyarsk), KY923065 (Kazhakstan), GU994784 (Mongolia) [28]).

Locality map and haplotype distribution map were constructed using ArcMap 9. 3 (ESRI, Redlands, California, USA) and Genetic Landscape Toolbox [29] and arranged in CorelDraw X5 (Ottawa, ON, Canada). Finally, haplotype rarefaction analysis was constructed using R3.2.3 [30] with the function *specaccum* of the *vegan* package [31].

## 3. Results

**Figure 1:**
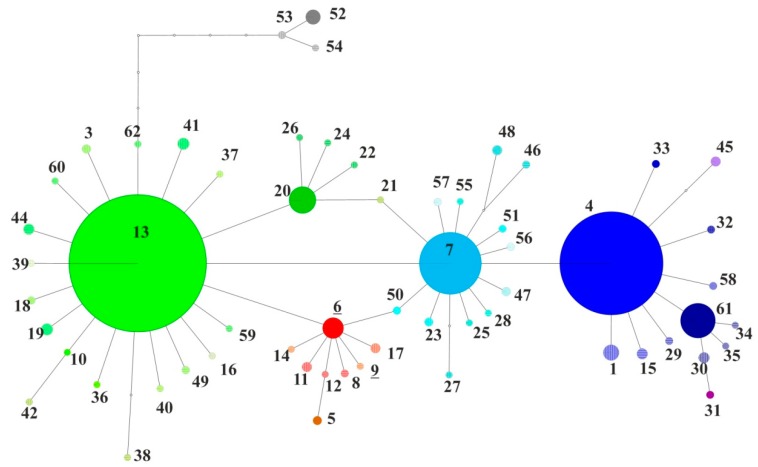
Parsimony network reconstructed by cladistic analysis with 95% connection limit using TCS 1.21. Numbers represent the haplotypes. Dimensions of pies are proportional to frequency, and each haplotype is colored based on its affiliation to a certain clade.

In total, 497 sequences 676 bp long (spanning from 699 to 1375 bp of *L. dispar* COI sequence) were used for further analyses. Cladistic analysis resulted in 60 haplotypes (Figure 1) distinguished by 25 singletons and 19 parsimony informative polymorphic sites, with the rarefaction curve of haplotypes reaching a plateau (data not shown) showing that the sampling effort was sufficient to describe intraspecific haplotype diversity [32]. Total haplotype diversity H_T_ was 0.81 and intrapopulation diversity H_S_ was 0.437 (data not shown). The lowest genetic diversity values were spotted in populations from the continental basin of the Balkan Peninsula between Dinaric Alps, Alps and Carpathian Alps and in northern Continental regions. On the other hand, the highest genetic diversity was observed in Coastal Croatia, Italy, and France. Populations from the Aegean coast and Black sea coast showed rather low diversity, while populations from Georgia showed intermediate values (Table 1).

**Figure 2:**
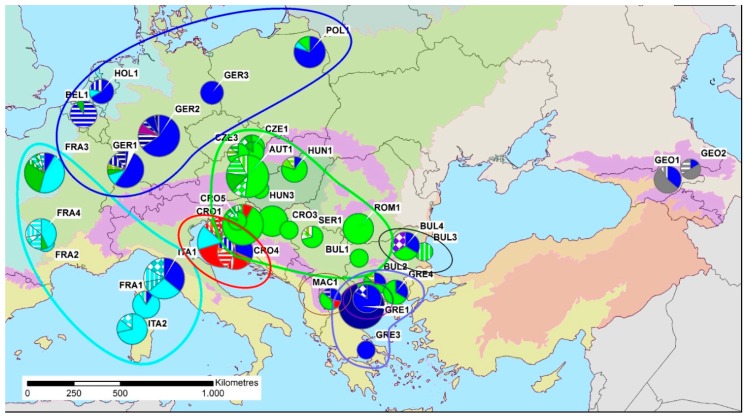
Haplotype distribution of *Lymantria dispar* in Europe. Colored circles represent the clades according to parsimony network (Figure 1), acronyms indicate localities (Table 1) and dimension of pies are proportional to sample size and haplotype abundance. Underlaying map represents biogeographic regions of Europe [33]. The figure was constructed using ArcMap 10.1 (ESRI, Redlands, CA, USA).

Fixation index G_ST_ (se) = 0.463 (0.056) was smaller than observed fixation index N_ST(obs.)_ (se) = 0.521 (0.059) which was significantly greater than theoretical fixation index N_ST(theor.)_ (se) = 0.456 (0.001) (*p* = 0.010), confirming the existence of a limited phylogeographic structure (G_ST_ < N_ST(obs.)_ > N_ST(theor.)_) (Figure 2). Nevertheless, AMOVA attributed not more than 24% of divergence to host selection (FCT=0.23831, *p* < 0.01), with the main source of divergence being found among populations within groups (FST = 0.53239, *p* < 0.01), and within populations (FSC = 0.38609, *p* < 0.01), rejecting the hypothesis of host-driven divergence at least among the broadleaved hosts analyzed in the current study. 

**Figure 3:**
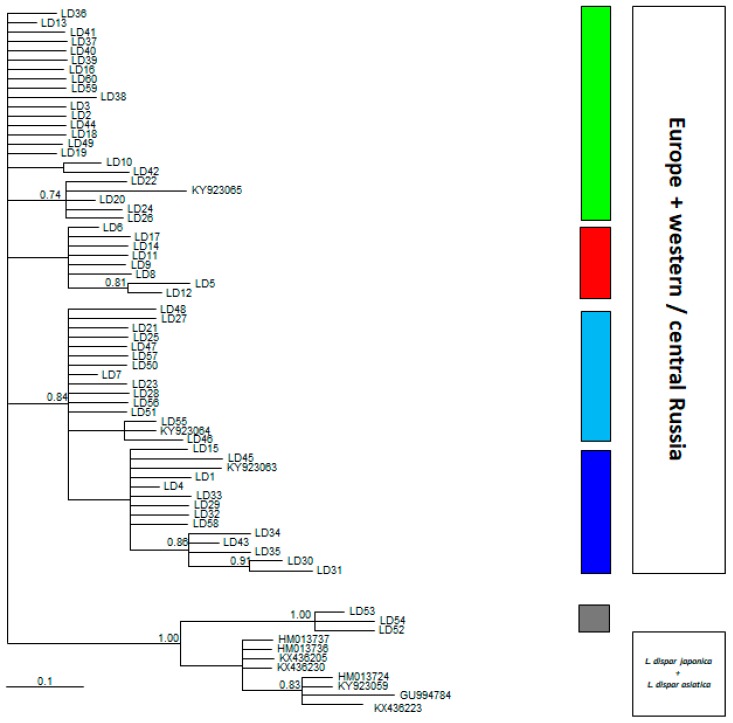
Bayesian reconstruction of molecular phylogenetic relationships among haplotypes of the current research supplemented with sequences submitted in GenBank (see text for detailed description). Numbers above nodes represent the Bayesian posterior probability with values greater than 0.6. Color of each bar is identical to the ones used in Figure 1.

The phylogenetic tree grouped European haplotypes in a similar pattern supporting the main clades with posterior probabilities higher than 80%, with the exception of green cluster that was the most common one in Central Europe. The inclusion of NCBI GenBank sequences from Asia, revealed the position of haplotypes LD52, LD53, and LD54 from Georgia diverged 1.46% from the European groups, while averagely diverged 0.86% from the cluster of Asian haplogroups *L. dispar asiatica*/*L. japonica* haplotypes (KX436223, KY923509, HM013724, GU994784, HM013737, HM013736, KX436205, KX436230) retrieved from NCBI (Figure 3). Finally, while Siberian sequences integrated in the European *L. dispar dispar* cluster, Georgian haplotypes formed a distinct clade, separated from *L. dispar japonica* and *L. dispar asiatica*.

## 4. Discussion

Gypsy moth is a holarctic species, flexible in terms of temperature tolerance [34] and it has extremely wide spectrum of host plants [35]. This species successfully invaded a vast variety of ecosystems, causing disturbances on a wide array of tree and general plant species. Model predictions for *L. dispar dispar* support a range shift of up to 900 km northwards in Europe as a consequence of increase in average air temperature [36]. Therefore, it is important to understand the phylogeography of *L. dispar dispar* in Europe regarding management strategies [37].

Major [38,39,40] and minor refugia [27,41] during ice ages coupled with recent gene flow events [42] determined the current pattern of divergence. Though for some species it has been demonstrated that the most recent ice age had the most profound impact on intraspecific diversity [43], for several other species the origin of divergence goes further back before the last ice age [44,45], with *Lymantria dispar* being one of them [13]. Intraspecific divergence among haplotypes of European *L. dispar dispar* (0.5029%) is congruent with previous findings that this clade arose around 200 thousand years (kyr) [13]. In particular, the observed genetic pattern of *L. dispar dispar* in Europe seems to have been shaped by at least two refugia, one located near the Carpathian Mountains (green) spreading to Central Europe and the Pontic-Mediterranean region (blue) spreading to the Balkans and the north of Europe. Furthermore, it is likely that contemporary pattern of gypsy moth phylogeography in Europe was also influenced by anthropogenic migration. This has been already hypothesized in the recent study of the Asian gypsy moth based on COI barcode sequences [46] that revealed an unusually close relationship between geographically distant haplotypes which could not be explained merely by natural dispersal of gypsy moth.

Being a highly polyphagous species, *L. dispar* populations would have been expected to exhibit deep intraspecific divergence as different selection pressures are associated with different host plants, something that favors the emergence of barriers to gene flow [47,48]. Should these barriers be maintained for a sufficiently long period of time, they may lead to the formation of distinct host-associated lineages [49]. This concept was initially thought to be valid mostly for parthenogenetically reproducing species [50,51,52], yet recent studies have shown that it is also evident in sexually reproducing species [49,53]. However, the limited genetic structure revealed among gypsy moth populations in Europe coupled with the AMOVA outcome do not support this expectation. As with other polyphagous insect species such as *Pityogenes chalcographus* [54] or *Heliconius melpomene* [55,56] life-history traits might have evolved on one host plant upon secondary contact could have been transmitted to populations feeding on a different host species, and thus render the herbivore insect species capable to feed on different host species [54]. Furthermore, this effect is reinforced by the high gene flow of *L. dispar* as the combination of the migratory pathways (flying moths, wind-borne dispersal of neonate larvae and long distances anthropogenic movement [9]) could facilitate the redistribution and coalescence of populations [57].

## 5. Conclusions

Despite the relatively large sample, we were able to determine only limited inferences of phylogeographic structure among localities in Europe, with at least two refugial areas contributing to the current genetic structure of gypsy moth. The near panmictic status of haplotype LD4 also present in Georgia suggests that more complex forces have taken role in shaping of the contemporary phylogeographic pattern of gypsy moth, and one of likely explanations could be a man-aided dispersal. Such hypotheses have already been drawn on larger scale studies [46]. However, the separation of the Georgian populations from other European populations, but also from the Asian haplotypes *L. dispar asiatica* and *L. dispar japonica*, shows that there are likely many more genotypic entities than recently described [28]. In fact, as differences in biology and behavior are described between gypsy moths from Europe and Asia, i.e., flight capability of females [10,58], the region of the Caucasus should be studied more thoroughly in order to examine the taxonomic status of the genetic entities in that geographic area.

## Figures and Tables

**Table 1:** Countries, localities, WGS84 coordinates in degrees (Lat., Long.), host tree (QE = *Quercus petraea*; QR = *Q. robur*; QI = *Q. ilex*; QP = *Q. pubescens*; QS = *Q. suber*; QO = *Q. coccifera*; FS = *Fagus sylvatica*; MA = *Malus* sp.; VAR1 = *Q.petraea* + *F.sylvatica*; VAR2 = *Q. robur* + *F. sylvatica*; VAR3 = *Q. robur* + *Q. petraea*; VAR4 = *Q. petraea* + *Q. pubescens*; VAR5 = *Salix* sp. + *Populus* sp.; VAR6 = *Q. rubra* + *Q. petraea* + *Q. cerris* + *Carpinus betulus* + *Fraxinus* sp.), number of individuals (N), number of haplotypes (HT), haplotype diversity (Hd), nucleotide diversity (π), mean number of pairwise differences (MNPD), and Allelic richness (r) after rarefaction to smallest sample size (5). Mean value and standard deviation (SD) is shown for all genetic diversity indices except for r.

Country	Abbrev.	Host	Lat.	Long.	N	HT	Hd ± SD	π ± SD	MNPD ± SD	r (5)
Austria	AUT1	QE	47.75	16.54	27	5	0.695 ± 0.078	0.001 ± 0.001	0.871 ± 0.632	1.966
Belgium	BEL1	FS	50.82	4.41	11	2	0.181 ± 0.143	0.001 ± 0.000	0.727 ± 0.584	0.455
Bulgaria	BUL1	QR	43.09	23.46	5	1	0 ± 0	0 ± 0	0 ± 0	0.000
	BUL2	QR	41.71	24.16	8	3	0.607 ± 0.164	0.002 ± 0.001	1.357 ± 0.933	1.518
	BUL3	QR	42.74	27.74	5	1	0 ± 0	0 ± 0	0 ± 0	0.000
	BUL4	QR	43.21	26.62	12	3	0.727 ± 0.058	0.002 ± 0.001	1.939 ± 1.181	1.788
Croatia	CRO1	QI	44.65	14.47	37	10	0.818 ± 0.038	0.003 ± 0.002	2.087 ± 1.193	2.555
	CRO2	QR	45.47	17.99	14	1	0 ± 0	0 ± 0	0 ± 0	0.000
	CRO3	QR	44.95	19.07	5	1	0 ± 0	0 ± 0	0 ± 0	0.000
	CRO4	QP	44.15	15.3	19	8	0.859 ± 0.052	0.004 ± 0.002	2.666 ± 1.485	2.815
	CRO5	VA1	45.22	16.28	25	6	0.426 ± 0.121	0.000 ± 0.000	0.473 ± 0.425	1.167
	CRO6	QR	45.65	15.59	6	2	0.333 ± 0.215	0.000 ± 0.000	0.333 ± 0.380	0.833
Czech Republic	CZE1	QE	49.11	17.04	10	4	0.533 ± 0.180	0.001 ± 0.001	0.600 ± 0.519	1.500
	CZE2	QR	48.88	17.1	7	2	0.285 ± 0.196	0.000 ± 0.000	0.285 ± 0.340	0.714
	CZE3	QE	48.85	16.01	7	3	0.523 ± 0.208	0.001 ± 0.001	0.857 ± 0.681	1.429
France	FRA1	QS	41.71	9.33	11	3	0.345 ± 0.172	0.000 ± 0.000	0.363 ± 0.377	0.909
	FRA2	QP	44.47	2.47	5	2	0.400 ± 0.237	0.000 ± 0.000	0.400 ± 0.435	1.000
	FRA3	QE	47.82	1.92	24	7	0.721 ± 0.070	0.002 ± 0.001	1.554 ± 0.961	2.038
	FRA4	QE	44.85	2.12	14	6	0.802 ± 0.090	0.001 ± 0.001	1.219 ± 0.824	2.500
Georgia	GEO1	MA	41.94	44.48	11	3	0.618 ± 0.103	0.008 ± 0.005	5.818 ± 3.013	1.407
	GEO2	MA	41.88	46.13	6	3	0.733 ± 0.155	0.005 ± 0.003	3.933 ± 2.290	1.833
Germany	GER1	VA2	48.26	7.76	20	6	0.636 ± 0.115	0.001 ± 0.001	1.194 ± 0.797	1.838
	GER2	VA3	49.91	10.19	26	6	0.566 ± 0.108	0.001 ± 0.001	1.141 ± 0.764	1.579
	GER3	QE	51.91	14.35	8	1	0 ± 0	0 ± 0	0 ± 0	0.000
Greece	GRE1	QO	40.77	23.11	31	3	0.127 ± 0.079	0.000 ± 0.000	0.193 ± 0.250	0.323
	GRE2	QO	41.08	23.54	12	2	0.303 ± 0.147	0.000 ± 0.000	0.303 ± 0.336	0.682
	GRE3	QO	38.63	22.97	5	1	0 ± 0	0 ± 0	0 ± 0	0.000
	GRE4	QO	41.12	25.41	9	2	0.500 ± 0.128	0.001 ± 0.001	1.000 ± 0.739	0.952
Holland	HOL1	QR	51.98	5.68	9	3	0.555 ± 0.165	0.000 ± 0.000	0.611 ± 0.530	1.389
Hungary	HUN1	VA4	47.83	19.96	10	4	0.533 ± 0.180	0.001 ± 0.001	0.800 ± 0.628	1.500
	HUN2	VA5	47.71	17.12	11	4	0.709 ± 0.099	0.001 ± 0.001	0.872 ± 0.661	1.851
	HUN3	VA6	47.07	17.34	5	2	0.600 ± 0.175	0.000 ± 0.000	0.600 ± 0.562	1.000
Italy	ITA1	QI	42.99	10.5	25	5	0.776 ± 0.046	0.002 ± 0.001	1.513 ± 0.941	2.245
	ITA2	QS	40.51	8.46	14	3	0.483 ± 0.142	0.000 ± 0.000	0.527 ± 0.467	1.209
FYROM	MAC1	N/A	41.36	21.21	7	4	0.809 ± 0.129	0.002 ± 0.001	1.714 ± 1.131	2.381
Poland	POL1	VA5	53.29	22.61	15	3	0.361 ± 0.144	0.001 ± 0.001	0.590 ± 0.500	0.905
Romania	ROM1	N/A	44.5	23.74	14	1	0 ± 0	0 ± 0	0 ± 0	0.000
Serbia	SER1	QE	44.47	5.57	7	3	0.523 ± 0.208	0.001 ± 0.001	0.857 ± 0.681	1.429
